# Incidence of Myelotoxicity and Other Adverse Effects Related to Thiopurine Starting in Patients with Inflammatory Bowel Disease: Retrospective Observational Study in a Third-Level Hospital

**DOI:** 10.3390/jcm12206571

**Published:** 2023-10-17

**Authors:** Gerard Grau, Eduard Brunet-Mas, Laura Patricia Llovet, Patricia Pedregal, Albert Villoria, Luigi Melcarne, Anna Puy, Belen Garcia-Sague, Luis Enrique Frisancho, María José Ramírez-Lázaro, Sergio Lario, Xavier Calvet

**Affiliations:** 1 Servicio Aparato Digestivo, Centro Médico Teknon, 08028 Barcelona, Spain; g.grau93@gmail.com; 2 Servei d’Aparell Digestiu, Parc Taulí Hospital Universitari, Institut d’Investigació i Innovació Parc Taulí (I3PT-CERCA), Departament de Medicina, Universitat Autònoma de Barcelona, 08207 Sabadell, Spain; eduardbrunet91@gmail.com (E.B.-M.); lpllovet@tauli.cat (L.P.L.); avilloria@tauli.cat (A.V.); lmelcarne@tauli.cat (L.M.); apuy@tauli.cat (A.P.); bgarcias@tauli.cat (B.G.-S.); lefrisancho@tauli.cat (L.E.F.); mjramirezl@tauli.cat (M.J.R.-L.); slario@tauli.cat (S.L.); 3 Centro de Investigación Biomédica en Red de Enfermedades Hepáticas y Digestivas (CIBERehd), Instituto de Salud Carlos III, 08035 Barcelona, Spain; 4 Servei de Gastroenterologia, Hospital de la Santa Creu i Sant Pau, Universitat Autònoma de Barcelona, 08193 Barcelona, Spain; patripedregal@gmail.com; 5 Departament de Medicina, Universitat Autònoma de Barcelona, 08193 Barcelona, Spain

**Keywords:** inflammatory bowel disease, thiopurines, azathioprine, mercaptopurine, adverse effects neutropenia, thiopurine methyltrasferase

## Abstract

Background and objectives: Thiopurines are an effective treatment for the maintenance of remission in inflammatory bowel disease (IBD). They can present adverse effects (AEs), with myelotoxicity being the most relevant. This study aims to determine the incidence of AEs related to the starting of thiopurines in our centre. Methodology: Retrospective study. The AEs in patients that were started on thiopurines between January 2016 and June 2020 were registered, with a two-year follow-up. The mean and standard deviation were used to describe the quantitative variables, and percentages and confidence intervals were used for the qualitative variables. The statistical significance was set at a *p*-value < 0.05. Results: 98 patients were included, with 64 AEs detected in 48 patients (49%). Most of the AEs appeared in the first 6 months. The most relevant were: 21 neutropenia (21.4%), 19 hypertransaminasemia (19.4%), 13 digestive intolerances (13.2%), 6 acute pancreatitis (6.12%), 3 phototoxicity (3%), and 2 unknown origin fevers (2%). In 29 patients (29.4%) the treatment had to be suspended due to AEs. In 11 cases (11.2%), azathioprine (AZA) was switched to 6-mercaptopurine (6 MP) as 5 showed tolerance and 6 patients needed suspension due to AEs. Eight patients required hospital admission, but none of them needed intensive care unit admission. There were no fatal adverse effects. Conclusions: Thiopurines are a safe drug with few AEs, especially after the first months of treatment. These results suggest that periodic analytic follow-up may not be necessary after the initial period of treatment.

## 1. Introduction

Inflammatory bowel disease (IBD) is a group of chronic, disabling, immune-mediated diseases affecting the gastrointestinal tract. The two most prevalent are ulcerative colitis (UC) and Crohn’s disease (CD) [1]. Their aetiology and pathogenesis are still not well understood. However, effective treatments are now available that allow us to modify the natural history of the disease [1]. 

One of the most widely used and effective treatments for IBD are the thiopurines, which include azathioprine (AZA) and 6-mercaptopurine (6-MP). They act by exerting a nonspecific antiproliferative and inhibitory effect on different cells of the immune system, especially T-lymphocytes, B-lymphocytes, and natural killer cells [2]. Thiopurines are indicated for the maintenance treatment of IBD in corticoresistant patients—patients who do not respond to steroids—or corticodependent patients—those who require continued treatment to maintain clinical remission [3]. Thiopurines in monotherapy maintain clinical remission with an efficacy of 70–80% [4]. Some studies have reported a mucosal healing rate of approximately 15–20% at 26 weeks [4,5].

Thiopurines are considered a reasonably safe drug, especially when compared to the risks of uncontrolled IBD. However, in up to 10–20% of patients, they have to be withdrawn due to toxicity [3]. There are two types of adverse effects (AEs): idiosyncratic (pancreatitis, arthralgia, and gastrointestinal intolerance), which occur at the start of treatment, usually within the first 3 months, and dose-dependent (myelotoxicity and hepatotoxicity), which can theoretically occur at any time, but usually within the first few months or years [2,6]. In addition, thiopurines can increase the risk of infection more than threefold (OR, 3.1; 95% CI, 1.7–5.5) [7] and the risk of malignancies, mainly lymphoproliferative syndromes, up to 5.3-fold [2.01–13.90; *p* = 0.0007]. They also increase the risk of non-melanoma skin tumours (IRR 1.64, 95% CI) and urinary tract tumours (HR 2.82 [95% CI, 1.04–7.68; *p* = 0.04]). The risk of malignancy increases markedly with age [2]. 

Regarding the thiopurine pathway, thiopurine methyltransferase (TPMT) is an enzyme responsible for the metabolization of both 6-mercaptopurine and 6-thioinosine monophosphate into their inactive metabolites. TPMT enzyme expression can be measured by TPMT activity in erythrocytes, identifying patients with high, low, or no TPMT activity. Patients with high TPMT activity (hypermethylators or “shunters”) use more of this enzyme pathway, leading to lower clinical efficacy and increased hepatotoxicity. Patients with decreased or absent TPMT activity metabolise the drug via the xanthine oxidase metabolic pathway (with production of the inactive metabolite 6-thiuric acid) and hypoxanthine-guanine phosphoribosyltransferase (HPRT), with the subsequent formation of 6-thioguanine nucleotides (6 TG) (active metabolites), leading to an increased risk of myelotoxicity [8].

The determination of TPMT enzyme activity allows the identification of patients with null activity in whom treatment is contraindicated due to the risk of severe myelotoxicity. Although TPMT determination is not fully established in clinical practice, it is recommended to be routinely applied to all patients as it is considered a cost-effective strategy [9]. 

Due to the significant rate of AEs, it is recommended to monitor blood counts and liver function in these patients. The recommendations of the Spanish working group on Crohn’s disease and ulcerative colitis (GETECCU) suggest a first check-up 15 days after starting treatment, a second check-up at one month, and a third check-up at two months, and thereafter check-ups every 4–6 months [3]. 

The aim of the study is to identify and quantify the main AEs (specifically neutropenia) that appear during the first two years of follow-up in patients treated with thiopurines in our centre and to analyse their impact on therapeutic management.

## 2. Materials and Methods

### 2.1. Design and Data Collection

A retrospective observational study of a cohort of patients identified by registration in the clinical history using the ICD-9-CM and the database of the inflammatory bowel disease unit of the Hospital Parc Taulí in Sabadell. 

### 2.2. Population

Patients with IBD who were started on treatment with thiopurines (monotherapy or with combined biological therapy) between 1 January 2016 and 30 June 2020, with a follow-up period of up to 2 years, were selected. The starting dose was 2 to 2.5 mg/kg, although in cases with low TPMT activity, patients may have been started at lower doses.

Patients who had not started treatment with thiopurines during the established period, who had abandoned follow-up in our centre, or who received such treatment for diseases other than IBD were excluded. 

### 2.3. Variables

Personal demographic variables (age, sex, and weight), disease variables (type of IBD), and thiopurine variables (TMPT activity, type of treatment, start date, dose, previous EBV immunity, and concomitant treatment with allopurinol) were collected. Treatment-related complications (acute pancreatitis, digestive intolerance, analytical alteration), analytical data at follow-up (blood count, transaminases, 6-TG and 6-MMP levels, lipase, and CRP), and disease evolution (clinical response, change of dose and/or treatment, and reason for change) were also registered. 

Normal TMPT activity was defined as greater than 14 U/mL by our laboratory. The sample was distributed in the following quartiles: TPMT < 14.8 (Q1), TMPT 14.8–17 (Q2), TPMT 17–19 (Q3), and TPMT 19–23.7 (Q4). Levels of 6-TG were in the sub-therapeutic range if below 230 U/mL, correct if 230–450 U/mL, and toxic if >450 U/mL. 6-MMP levels were defined as in range if below 5700 U/mL and toxic if above this value.

Neutropenia was present if the neutrophil count was less than 2 × 109/L; it was classified as mild with values between 1.5–2 × 109/L, moderate 1–1.5 × 109/L, and severe if the neutrophil count was less than 1 × 109/L. Anaemia was determined by haemoglobin values <130 g/L in men and <120 g/L in women. Thrombocytopenia was present if platelets were below 150,000/µL, being mild between 100,000/µL and 150,000/µL, moderate between 50,000/µL and 99,999/µL, and severe below 50,000/µL. Normal transaminase values were determined between 0–32 U/L for aspartate aminotransferase (AST) and 10–31 U/L for alanine aminotransferase (ALT), with moderate hypertransaminasemia being considered an elevation above twice the upper normal value and severe hypertransaminasemia four times higher (AST > 128 and ALT > 124). C-reactive protein (CRP) was considered normal below 0.5 mg/dL.

### 2.4. Statistical Analysis

For all quantitative variables, the mean and standard deviation were calculated. For qualitative variables, percentages with their 95% confidence intervals were calculated. The Mann-Whitney test was used to compare independent variables. Fisher’s exact test was used to compare the proportions between the groups. A subanalysis regarding patients older than 65 was performed; major findings were compared with those of younger patients. The level of statistical significance was set at 0.05. 

### 2.5. Ethical Aspects

All data were compiled in an Excel database with no patient identifiers. Only a restricted access list with the patient’s history number was made available to the researchers.

The study was reviewed and approved by the local ethics committee (CEIM 2022/5045). Given its retrospective nature, with no impact on the patient’s evolution or treatment, it was not considered necessary to obtain informed consent. The study was conducted in compliance with the requirements of the Declaration of Helsinki [10]. 

## 3. Results

A total of 98 patients were identified; 49 were female (50%), and the mean age at baseline was 43.3 years (range 17–82). Regarding the type of inflammatory disease, 58 patients were identified with CD, 38 with UC, and 2 with indeterminate colitis (59%, 39%, and 2%, respectively). The mean follow-up time was 517 days (range 4–846) (see Table 1 for details).

**Figure 1:**
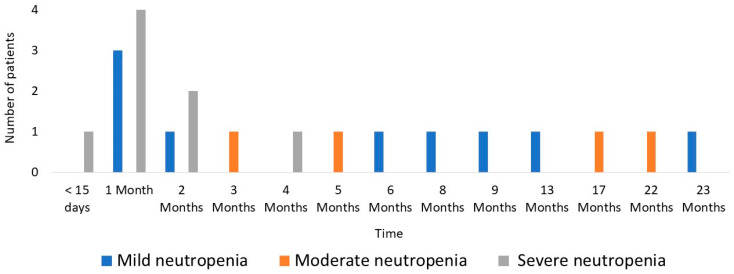
Chronology of neutropenia onset.

A total of 64 AEs were recorded in 48 patients (49%). Adverse events were as follows: 21 neutropenia (21.4%) (9 mild, 4 moderate, 8 severe), 19 hypertransaminasemia (19.4%) (17 mild, 1 moderate, 1 severe), 13 digestive intolerances (13.2%), 6 acute pancreatitis (6.1%), 3 phototoxicity (3%), and 2 fevers of unknown origin (2%). Two patients experienced an AEs before the first analytical follow-up (acute pancreatitis in both cases). Figure 1 shows the chronology of the onset of neutropenia during the follow-up period. 80.9% (*n* = 17) of the cases of neutropenia appeared in the first 9 months. No severe anaemia was detected beyond 4 months after the start of treatment (see Figure 1 for details).

**Figure 2:**
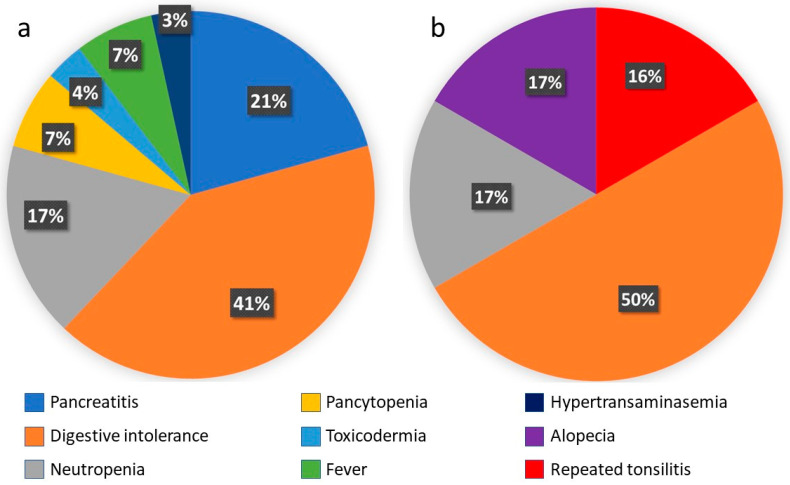
(**a**) Causes of azathioprine suspension. (**b**) Causes of 6MP suspension. (Number of cases; % of total discontinuations).

Azathioprine treatment had to be discontinued in 29.6% of cases (*n* = 29). The main reasons for discontinuation were all cases of acute pancreatitis and digestive intolerance and most cases of severe neutropenia cases (87.5% (*n* = 7); in the remaining 12.5% (*n* = 1), treatment could be reintroduced at lower doses). There were two cases of pancytopenia: one included in the severe neutropenia group, this being the most severely affected line, and another with mild involvement of the white series, highlighting severe central anaemia. Other causes of withdrawal were severe hypertransaminasemia (one patient, 5.3% of cases of hypertransaminasemia), toxicodermia (one patient, 33% of cases of skin toxicity), and fever of unknown origin (two patients) (see Figure 2a for details). No subjects in our study stopped thiopurines because of a lack of efficacy. In cases of lack or loss of efficacy, most patients started combined treatment with an anti-TNF drug. In this case, the thiopurine dose may have been decreased but not stopped. There were no deaths associated with any AEs.

In 11 patients, thiopurine was switched from azathioprine to mercaptopurine (9 due to digestive intolerance, 1 after an episode of fever of unknown origin, and 1 due to toxicodermia). Among these, adverse effects of mercaptopurine were detected in six patients (54.5%); all of them led to discontinuation of the drug, including three patients due to persistent digestive intolerance (50%), one case of severe neutropenia (16.6%), one case of recurrent tonsillitis (16.6%), and one case of alopecia (17%) (see Figure 2b for details).

A subanalysis including 16 patients older than 65 years (16.3%) showed that 10 patients (62.5%) presented a total of 11 AEs (68.8%), and seven patients discontinued the treatment (43.8%). Comparing this subgroup of elder patients with the younger patients, no differences were observed regarding AEs (68.8% vs. 46.3%; *p* = 0.13), and significant statistical differences were observed in the discontinuation of the treatment (43.8% vs. 26.9%; *p* < 0.001).

Regarding TPMT activity levels, no statistically significant differences were found between patients with and without neutropenia (17.56 vs. 16.92; *p* = 0.21). Only 5 out of 21 patients with neutropenia (23.8%) were in the lower quartile in terms of TPMT activity value. Likewise, no differences were found for patients with severe neutropenia. Table 2 shows detailed information about the patients with detected neutropenia during follow-up.

## 4. Discussion

Our study shows that the main effects detected were myelotoxicity, acute pancreatitis, skin conditions, altered liver function, and digestive intolerance. It should be noted that the occurrence of severe neutropenia was practically non-existent 6 months after the start of treatment.

In the literature, the incidence of leukopenia (used as the main marker of myelotoxicity) is approximately 3% per patient per year of follow-up, with a cumulative incidence of 7% reported [11]. These data are lower than those detected in our series, where the occurrence of neutropenia was 21%. This may be explained by the fact that the presence of neutropenia is not always accompanied by absolute leukopenia, so studies evaluating this parameter may be underestimating the prevalence of myelotoxicity. In our study, 5 of the 21 cases of neutropenia had a leukocyte count within the normal range. In addition, differences between studies are also due to the disparity between cut-off points between countries and even between hospitals and laboratories for defining pathological values. In the systematic review by Gisbert J.P. et al. [11], the cut-off point for neutropenia was generally found to be 1.5 × 109/L, lower than in the present study, which was 2 × 109/L. In our study, eight cases of severe neutropenia (8.2%) were detected, in most cases leading to the discontinuation of treatment. The data are higher than those reported in the literature, where severe leukopenia rates of up to 5.7% are defined [12,13,14]. These differences may be due to the sample size of the studies (8300 patients in a systematic review of 66 studies of Gisbert J.P. et al. vs. 98 in our study). Although myelotoxicity can occur at any time after drug initiation, the literature describes that it mostly occurs after the first weeks or months of treatment [11]. These data are consistent with those obtained in our study, where most neutropenia cases were recorded in the first nine months of treatment and no severe neutropenia was observed beyond 5 months.

Despite the evidence and recommendations to analyse TPMT activity prior to the initiation of a thiopurine [9,15,16], in our study no statistically significant correlation was found between the appearance of neutropenia during follow-up and TPMT activity levels. This could be explained by a selection bias, as all patients had been elected to start treatment with thiopurines after verifying normal TMPT activity, not including those patients that had been rejected to start treatment with thiopurines due to low activity.

Regarding the rest of the described AEs, the rate of acute pancreatitis detected was similar to what is described in the literature (7.3%) [9,11]. All cases of acute pancreatitis were mild and resolved after drug withdrawal, and in no case was thiopurine treatment re-started.

The prevalence of digestive intolerance was also similar to that described in other studies, as, for example, in this same study by Chisick et al. [9], where 62 out of 510 (12.1%) discontinued treatment due to the onset of nausea not associated with pancreatitis.

In this regard, it should be noted that although myelotoxicity is the most relevant adverse effect, both in our study and in routine clinical practice, digestive intolerance is the most frequent adverse effect in terms of causes of treatment suspension.

Almost one-third of the patients initially treated with azathioprine stopped treatment. These results are comparable to the discontinuation rates published in the study by Neils Teich et al. [17], where 186 out of 510 (36.4%) patients discontinued treatment. This high discontinuation rate may be due to, on the one hand, the availability of alternatives to thiopurines and, on the other hand, the fact that there are no clearly established indications as to which AEs and what level of severity should lead to discontinuation of the drug or the conditions that would allow reintroduction at standard or lower doses, leaving the decision to the physician. Other studies reported a higher rate of discontinuation; in the study of Macaluso FS et al., the rate of discontinuation was 63.2%, mostly due to gastrointestinal symptoms. More interestingly, the switch to 6-MP was ineffective in 79.5% of patients [18].

In this reported series, AEs were detected in almost half of the patients treated with thiopurines, although there were no fatal events. Costantino et al. described that almost half of AEs appear very early (first 6 months), while late AEs (within 6 months) are less frequent [19].

Regarding elderly patients, 62.5% of patients presented AEs and 43.8% discontinued the treatment. Calafat et al. reported that the rate of discontinuation is higher in elderly patients than in young patients (67.2% vs. 63.1%; *p* < 0.001) [6]. Despite the fact that in both studies the discontinuation of thiopurine treatment in elderly patients is statistically significant, our rates are substantially lower, probably due to the limited number of elderly patients registered.

These results lead us to see thiopurines as a group of drugs that, apart from their proven clinical efficacy, are safe if there is a good understanding of their possible AEs and how to deal with them. It should be noted that this study contemplated a follow-up of up to 2 years, making the detection of long-term AEs, such as neoplasms, less likely. In addition, the sample had a mean age at the start of treatment of 43.3 years, making the development of malignancies even less likely.

The results of this study are comparable to the findings of other larger reviews. Our data support previous recommendations regarding the importance of closer clinical and analytical follow-up in the first few months and being subsequently laxer if no incidences are detected.

Thus, because of this study, we propose a first check-up around 15 days after the start of treatment. A second control 4 weeks after the previous one (in case of TMPT < 14, 6 TG and 6 MMP levels will be requested, individualising subsequent controls according to the results) and a third control 3 months after the previous control and subsequently every 3 months until the first year is completed, extending at 6-month intervals thereafter.

Although previous studies have discouraged the cessation of monitoring because of the possibility of myelotoxicity anytime during follow-up, the extremely low incidence of these events after the first few months could call into question the cost-effectiveness of such monitoring.

The study has several limitations. Firstly, its retrospective nature, which does not allow for a prospective design with unanimity of criteria and rigorous data collection, for example, the lack of weight data did not allow us to give exact information on starting doses adjusted per kilogram. In addition, the size of the sample, the disparity of criteria in the management of therapeutic adjustment, and the attitude of the different physicians involved towards the diverse AEs limited the strength of the study.

In conclusion, the low incidence of severe AEs in our study after the first months, together with previous data described, suggests that such strict periodic controls may not be cost-effective. In fact, most IBD units have moved from three- or four-monthly to six-monthly monitoring in recent years, following the recommendations of the latest ECCO guidelines. In this regard, it would be interesting to evaluate in subsequent studies the possibility of extending the periods between analytical controls, for example, to an annual determination or even to perform them on demand according to the appearance of symptoms.

## Figures and Tables

**Table 1:** Sample population characteristics.

Variables		% (*n*)
Gender	Men	50% (*n* = 49)
	Women	50% (*n* = 49)
IBD Type	Crohn’s disease	59.2% (*n* = 58)
	Ulcerative colitis	38.8% (*n* = 38)
	Indeterminate colitis	2% (*n* = 2)
Mean age at treatment start		43.3 years (17–82)
Mean follow-up time		517 days (4–846)
Mean TPMT activity		17.06 (10–23.7)
EBV IgG +		98% (*n* = 96)

**Table 2:** Detailed neutropenia cases. Age, gender, leucocyte count, type of IBD, and time to event.

Age	Gender	IBD Type	TPMT	Leucocytes (×10^9^/L)	Neutrophiles (×10^9^/L)	Time to AEs (Days)	Time to AEs (Months)
81	Male	UC	13.9	2.54	0.86	32	1.1
21	Male	UC	18.8	2.01	0.82	37	1.2
30	Female	CD	18.3	4.85	1.81	189	6.2
42	Male	UC	18.8	3.57	1.33	105	3.5
77	Female	CD	17.6	2.05	0.03	62	2.0
30	Male	CD	19.2	2.86	1.72	700	23.0
20	Male	UC	18.1	1.93	0.84	47	1.5
70	Female	UC	19.2	3.84	1.93	409	13.4
50	Female	CD	18.5	3.49	1.68	28	0.9
26	Male	UC	19.9	3.56	1.85	42	1.4
35	Male	UC	18.9	1.51	0.39	34	1.1
65	Female	CD	12.5	3.03	1.79	64	2.1
25	Female	CD	13.5	3.19	1.31	662	21.8
82	Male	UC	13	3.39	1.79	23	0.8
49	Female	CD	22.1	3.71	1.8	272	8.9
58	Male	CD	19.6	3.22	1.98	234	7.7
43	Female	UC	17.9	2.97	0.87	7	0.2
60	Female	UC	13.6	1.65	0.41	53	1.7
25	Male	UC	16.7	2.62	1.18	140	4.6
62	Female	CD	19.8	1.58	0.94	108	3.6
39	Female	CD	18.2	2.95	1.47	507	16.7

## Data Availability

The data extraction template is available upon reasonable request.
